# 

**Published:** 1907-09

**Authors:** 


*The following donations have been received since the publication of the List in June:*

*August 31st, 1907.*

| | |
|---|---|
| L. M. Griffiths (1) | 1 volume. |
| Middlesex Hospital (2) | 1 ,, |
| R. Shingleton Smith, M.D. (3) | 5 volumes. |

Unbound periodicals have been received from Dr. Shingleton Smith, and pamphlets have been presented by Mr. L. M. Griffiths.

### SIXTY-FIFTH LIST OF BOOKS.

The titles of books mentioned in previous lists are not repeated.

The figures in brackets refer to the figures after the names of the donors and show by whom the volumes were presented. The books to which no such figures are attached have either been bought from the Library Fund, or received through the *Journal*.

**Adamson, H. G.** .. *Skin Affections of Childhood* .. .. .. .. 1907
**Ballance, C. A.** .. *Some Points in the Surgery of the Brain* .. 1907
**Barwell, H.** .. .. *Diseases of the Larynx* .. .. .. .. .. 1907
**Bennie, P. B.** .. *Treatment of Hip Disease* .. .. .. .. 1907
**Budin, P.** .. .. *The Nursling.* (Tr. by W. J. Maloney) .. 1907
**Campbell, H.** .. *On Treatment* .. .. .. .. .. .. .. 1907
**Ehrlich, P.** .. .. *Collected Studies on Immunity.* (Tr. by C. Bolduan) .. .. .. .. .. .. .. 1906
**Guthrie, L. G.** .. *Functional Nervous Disorders in Childhood* 1907
**Horsley and Mary D. Sturge, Sir V.** *Alcohol and the Human Body* 1907
**Janet, P.** .. .. .. *The Major Symptoms of Hysteria* .. .. 1907
**Johnson, J.** .. .. *Change of Air* .. .. .. (1) 3rd Ed. 1832
**Lowe, P.** .. .. *A Discourse on the Whole Art of Chyrurgery* (3) 4th Ed. 1654
**Luff, A. P.** .. .. *Gout* .. .. .. .. .. .. .. .. 3rd Ed. 1907
**Madden, F. C.** .. *Bilharziosis* .. .. .. .. .. .. .. .. 1907
**Maddox, E. E.** .. *Ophthalmological Prisms* .. 5th Ed. 1907
**Miles, A. Thomson and A.** *Manual of Surgery* Vol. II. 2nd Ed. 1907
**Murrell, W.** .. *What to do in Cases of Poisoning* 10th Ed. 1907
**Oppenheimer, C.** .. *Toxines and Anti-Toxines.* (Tr. by C. A. Mitchell) .. .. .. .. .. .. .. .. 1906
**Osler, W.** .. .. *The Growth of Truth* .. .. .. .. (3) 1907
*Pharmacopœia of the Bristol Royal Hospital for Sick Children and Women* .. .. .. .. .. .. .. .. 1907
**Poynton, F. J.** .. *Heart Disease and Thoracic Aneurysm* .. 1907
**Sargent, P.** .. .. *Surgical Emergencies* .. .. .. .. .. 1907
**Scott, H. H.** .. .. *Post-Graduate Clinical Studies.* 1st Ser. 1907
**Starling, E. H.** .. *The Croonian Lectures* .. .. .. .. (3) 1905
**Startin, J.** (Ed.) .. *A Skin Pharmacopœia* .. .. .. 6th Ed. 1907
**Steell, G.** .. .. *Diseases of the Heart* .. .. .. .. .. 1907
**Sturge, Sir V. Horsley and Mary D.** *Alcohol and the Human Body* 1907
**Sutherland, G. A.** *Treatment of Disease in Children* .. .. .. 1907
**Sylvius, F. de la B.** *Praxeos Medicæ* .. .. (3) *Editio altera* 1672
**Thomson and A. Miles, A.** *Manual of Surgery.* Vol. II. 2nd Ed. 1907

### TRANSACTIONS, REPORTS, JOURNALS, &c.

American Dermatological Association, Transactions of the .. .. 1906
American Journal of Ophthalmology, The .. .. Vol. XXIII. 1906
Archives of Neurology .. .. .. .. .. .. Vols. II., III. 1903–07

| | | |
|---|---|---|
| Archives of the Middlesex Hospital .. .. .. | (2) Vol. IX. | 1907 |
| Bäder-Almanach .. .. .. .. .. .. .. .. .. .. .. .. .. | (3) | 1907 |
| Bristol Port Sanitary District, Annual Report of the Medical Officers of the, 1906 .. .. .. .. .. .. .. .. .. .. .. | | 1907 |
| British Medical Journal, The .. .. .. .. .. .. | Vol. I. for | 1907 |
| Clinical Journal, The .. .. .. .. .. .. .. .. | Vol. XXIX. | 1907 |
| Edinburgh Medical Journal, The .. .. | N.S., Vol. XXI. | 1907 |
| English Catalogue of Books, The, for 1906.. .. .. .. .. .. .. | | 1907 |
| Hospitals— | | |
| St. Thomas's Hospital Reports .. .. .. | Vol. XXXIV. | 1906 |
| Westminster Hospital Reports .. .. .. .. .. | Vol. XV. | 1907 |
| Journal of Laryngology, The .. .. .. .. .. .. | Vol. XXI. | 1906 |
| Journal of Medical Research, The .. .. .. .. | Vol. XV. | 1906 |
| Lancet, The .. .. .. .. .. .. .. .. .. .. .. .. | Vol. I. for | 1907 |
| Library Association Record, The .. .. .. .. | Vol. VIII. | 1906 |
| Münchener medizinische Wochenschrift .. .. .. .. | Bd. I. | 1907 |
| Obstetrical Transactions .. .. .. .. .. .. | Vol. XLVIII. | 1907 |
| Progressive Medicine .. .. .. .. .. .. .. .. .. .. | Vol. II. | 1907 |

### MEETINGS OF SOCIETIES.

### Bristol Medico-Chirurgical Society.

### Local Medical Notes.

**University College, Bristol.**—Examination Results :—

M.B. LOND.—*Intermediate Examination :* P. J. Veale.

F.R.C.S.—*Primary Examination :* E. A. Dorrell.

CONJOINT BOARD.—*Practical Pharmacy :* H. H. Hiley, V. Pinnock. *Chemistry and Physics :* H. B. Logan, G. H. Piercy, W. Worger, B. G. Derry, A. G. T. Fisher. *Anatomy and Physiology :* G. H. Griffiths, W. A. Reynolds, M. M. Lopez, R. S. S Statham, H. R. B. Hull. *Medicine :* F. T. Boucher, A. E. Iles*. *Surgery :* F. S. Scott.

* Completes Examination.

L.D.S. Eng.—*Chemistry and Physics:* L. J. Kinnersley, J. D. Melhuish. *Physics only:* G. Smith.

Indian Medical Service.—At the recent examination for appointments in the Indian Medical Service, three students of University College, Bristol, were successful. Mr. V. B. Green-Armytage gaining second place, with 3,834 marks; Mr. Francis Shingleton Smith, M.B. Cantab., tenth place, with 3,410 marks; and Mr. A. N. Thomas, fourteenth place, with 3,283 marks. There were thirty-four candidates for the examination, and of these twenty-seven qualified to sit for the examination.

Dental Department.—It is now possible for students to undertake their Mechanical Dentistry and Metallurgy on the College premises, where laboratories have been fitted out with all the latest apparati for this purpose. The composition fee for the entire curriculum, including Hospital practice and *two* years' mechanical work in the laboratory, is 140 guineas.

The following appointments have recently been made:—Carey F. Coombs, M.D., B.S. Lond., Demonstrator in Dental Bacteriology; E. A. G. Dowling, L.R.C.P., M.R.C.S., L.D.S., Lecturer in Dental Anatomy, Physiology, and Dental Histology; Frederick W. Perry, L.D.S., Demonstrator in Dental Anatomy, Physiology and Dental Histology; J. W. McBain, M.A., Lecturer in Dental Metallurgy and Practical Dental Metallurgy; Reginald Davis, Instructor in Dental Mechanics; W. J. Lennox, L.D.S. Eng., Honorary Demonstrator in Physiology.

Admission of Women Students to Medical Department.—We understand that the Council of the College has undertaken the important step of admitting women to the full medical curriculum. Previously they had been allowed to attend certain lectures, but this privilege had not to any great extent been taken advantage of. The rules now admit them to the whole course on exactly the same footing as men.

**Bristol General Hospital.**—J. Odery Symes, M.D. Lond., has been appointed Physician, and Carey F. Coombs, M.D. Lond., has been appointed Assistant Physician.

**Dr. John Beddoe.**—An interesting ceremony took place recently at the Bristol Art Gallery, when a portrait of Dr. Beddoe, by Miss Wren, was handed over to the chairman of the Museum Committee for hanging in the Bristol Room. It had been subscribed for by Dr. Beddoe's friends and admirers, who wished to have some permanent momento of this great ethnologist, who had lived and worked in the city for so many years. Dr. Beddoe, who was present, thanked the speakers for their kind expressions of affection.

